# Epidermal Fatty Acid-Binding Protein: A Novel Marker in the Diagnosis of Dry Eye Disease in Sjögren Syndrome

**DOI:** 10.3390/ijms19113463

**Published:** 2018-11-04

**Authors:** Megumi Shinzawa, Murat Dogru, Seika Den, Takehiro Ichijima, Kazunari Higa, Takashi Kojima, Noriyuki Seta, Takeshi Nomura, Kazuo Tsubota, Jun Shimazaki

**Affiliations:** 1 Department of Ophthalmology, Tokyo Dental College, Ichikawa General Hospital, Chiba 2728513, Japan; mshinz2003@yahoo.co.jp (M.S.); denseika@gmail.com (S.D.); higakazunari@tdc.ac.jp (K.H.); kojkoj@me.com (T.K.); jun@eyebank.or.jp (J.S.); 2 Department of Ophthalmology, Keio University School of Medicine, Tokyo 1608582, Japan; tsubota@eyebank.or.jp; 3 Department of Dental and Oral Surgery, Tokyo Dental College, Ichikawa General Hospital, Chiba 2728513, Japan; ichijimatakehiro@gmail.com (T.I.); tanomura@tdc.ac.jp (T.N.); 4 Department of Internal Medicine, Tokyo Dental College, Ichikawa General Hospital, 2728513 Chiba, Japan; nseta@tdc.ac.jp

**Keywords:** epidermal fatty-acid binding protein (E-FABP), Sjögren’s syndrome, dry eye, tears

## Abstract

Purpose: Sjögren syndrome (SS) is a chronic inflammatory autoimmune disease of the lacrimal and salivary glands. This study compared the concentrations of epidermal fatty-acid binding protein (E-FABP) in the saliva, serum, and tears of SS patients with dry eye and dry mouth, with those of healthy adults to investigate the usefulness of E-FABP as a diagnostic marker for SS. Design: Prospective, observational case series. Participants: The subjects were 11 new patients with untreated Sjogren syndrome and 12 healthy control individuals. Methods: The diagnosis of SS was in accordance with the Ministry of Health, Labour and Welfare (Japan) Diagnostic Criteria (1999). Saliva, serum, and tear specimens were collected during internal medicine, dental, and ophthalmological examinations. The ophthalmological tests included the Dry Eye-related Quality of life Score (DEQS), tear break-up time (BUT), vital staining with fluorescein (FS) and lissamine green (LG), and the Schirmer test-1. The E-FABP concentration in the tears, saliva, and serum was measured by enzyme-linked immunosorbent assay (ELISA). Main outcome measure: The E-FABP concentrations were compared between patients and controls. Results: There were significant differences between the patient and healthy control groups in all ophthalmological test results. There were no significant differences between the groups in the E-FABP concentrations in the saliva (*p* = 0.1513) or the serum (*p* = 0.4799), but the E-FABP concentration in the tears significantly differed between groups. The E-FABP concentration in tears tended to be significantly lower in patients with SS (mean, 323.5 ± 325.6 pg/mL) than healthy control subjects (mean, 4076 pg/mL; *p* = 0.0136). The E-FABP concentration in tears significantly correlated with the results of dry eye parameters. Conclusion: The E-FABP concentration in tears appears to be related to ocular surface epithelial damage and tear stability and may be a promising novel biomarker in the diagnosis of SS.

## 1. Introduction

Sjögren syndrome (SS) is a chronic inflammatory autoimmune disease of the lacrimal and salivary glands [1]. According to the 2011 Survey on Autoimmune Diseases issued by the Research Project on Policy for Intractable and Other Diseases (Ministry of Health, Labour and Welfare-Japan Grants-in-Aid for Scientific Research) during fiscal year 2011, 68,483 patients with SS were examined in medical facilities in Japan; the prevalence of the disease was 0.05%, and the percentage of primary and secondary SS was 58.5% and 39.2%, respectively. According to the same report, when latent cases are included, the estimated number of patients in Japan is thought to be between 100,000 and 300,000 [2]. In the United States, SS is the second most common autoimmune disease, affecting nearly four million Americans, with an estimated prevalence of 0.5–5% [3]. The diagnostic criteria for SS were standardized in Japan in the Revised Diagnostic Criteria (JPN; Ministry of Health, Labour and Welfare, 1999) (Table 1) [4]. Other criteria include the American–European Consensus Group (AECG) criteria [5] and the American College of Rheumatology (ACR) criteria [6], which were recommended by the Sjögren’s International Collaborative Clinical Alliance (SICCA) as the international standard in 2012. Among the ophthalmological tests included in the ACR criteria is the use of the SICCA Ocular Staining Score (OSS). The degree of severity revealed by this test is significantly correlated with the results of systemic tests, such as those for the rheumatoid factor, antinuclear antibody titer, and immunoglobulin G level, which suggests that ocular severity is an important factor for the diagnosis of SS [7].

Because ophthalmological testing forms the basis for the diagnosis of SS, which presents with a variety of systemic symptoms, ophthalmologists play a major role in the diagnosis of the disease. Previous research into the mechanism of onset of SS identified the appearance of multiple autoantibodies, as well as the presence of autoreactive lymphocytes infiltrating organs, and as a result it is thought that an autoimmune response is the cause of the disease. However, as the cause remains poorly understood, continuous research is being conducted for a method of early diagnosis and markers that are specific to disease activity.

We have recently shown oxidative stress induced lacrimal gland and keratoconjunctival damage in patients with SS [8]. Recently, the fatty-acid binding protein (FABP) family has been reported as a novel marker that is useful in the diagnosis of diseases associated with oxidative stress, such as acute renal failure, myocardial infarction, and Alzheimer’s disease [9,10,11]. Currently, the national health insurance system in Japan covers L (Liver)-FABP (FABP1) as a diagnostic marker for acute and chronic renal failure and H (Heart)-FABP (FABP3) as a diagnostic marker for acute myocardial infarction. FABP forms a molecular family, and although there are a variety of expressions depending upon the specific organ or cell type, at least nine molecular species have been identified, and FABP12 has been recently discovered [12]. The FABP molecule acts as an intracellular chaperone for long-chain unsaturated fatty acids, and as such, it binds to insoluble long-chain fatty acids such as arachidonic acid and docosahexaenoic acid (DHA). It is thought to be involved in a variety of intracellular functions because it makes these fatty acids soluble [13,14,15]. Since our previous research suggested that the accumulation of lipid peroxidation products causes damage to the lacrimal glands in patients with SS, we decided to focus on the role of E-FABP (FABP5), which is expressed in the oral mucosa, ocular surface, and exocrine glands as a diagnostic marker for dry eyes in patients with SS in the current study.

## 2. Results

### 2.1. Subjects and Examinations

This was an observational case series study of 11 newly diagnosed patients with untreated dry eye or dry mouth (1 male, 10 females; age range: 37–74 years) who were examined at Tokyo Dental College Ichikawa General Hospital and 12 healthy control subjects (3 males, 9 females; age range: 37–75 years). The study was approved by the institutional ethics review board of Tokyo Dental College Ichikawa General Hospital (I 15−89, 10 February 2016) and was registered at University Hospital Medical Information Network (UMIN) Center. Written informed consent was obtained. The subjects in the patient group underwent the same diagnostic testing protocol at the departments of ophthalmology, internal medicine, and dental and oral surgery; their diagnoses of SS were conducted in accordance with the Japanese Ministry of Health, Labour and Welfare (MLHW) Revised Diagnostic Criteria (1999) (Table 1). No statistically significant differences were found between the patient and healthy control groups in terms of age (*p* = 0.0812 by Mann–Whitney U test) or sex ratio (*p* = 0.5901 by Fisher’s exact test).

### 2.2. Fatty Acid-Binding Protein Concentration in Tears, Saliva, and Serum Measured by Enzyme-Linked Immunosorbent Assay

**Figure 1:**
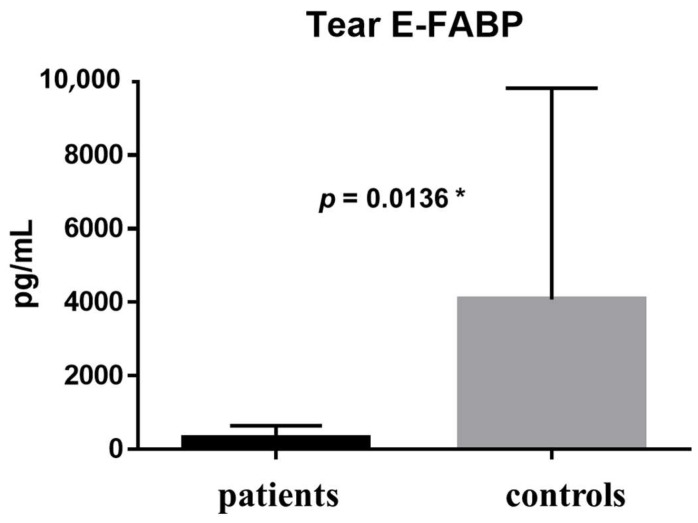
Tear E-FABP concentrations. E-FABP concentration in tears was 323.5 ± 325.6 pg/mL (range: 28.08–710.5 pg/mL; median: 169.1 pg/mL)in the patient group (*n* = 5) and 4076 ± 5746 pg/mL (range: 106.3–18516 pg/mL; median: 1629 pg/mL) in the control group (*n* = 12); the difference between the groups was statistically significant (*p* = 0.0136). Data are expressed as the mean with SD (one bar per column).

**Figure 2:**
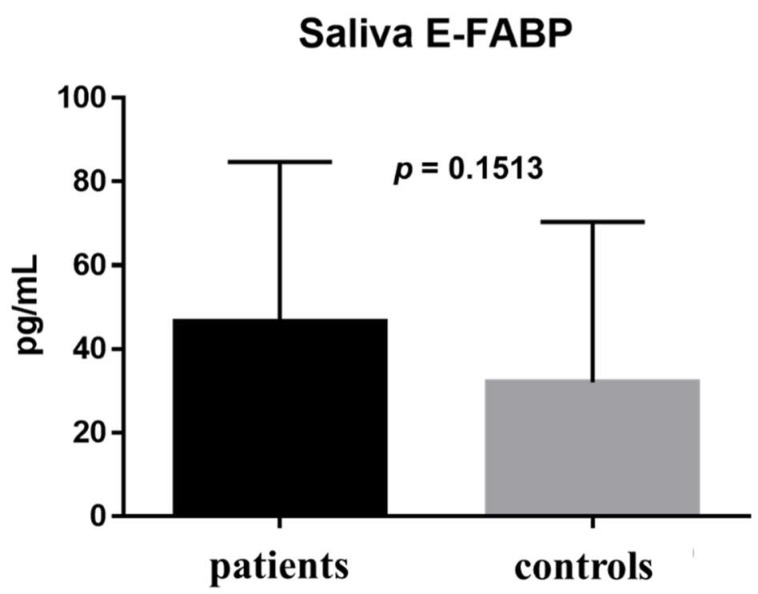
Saliva E-FABP concentrations. The E-FABP concentration in the saliva was 46.46 ± 38.22 pg/mL (range: 8.527–130.6 pg/mL; median: 40.44 pg/mL) in the patient group (*n* = 9) and 32.08 ± 38.31 pg/mL (range: 6.1–107.3 pg/mL; median: 20.38 pg/mL) in the control group (*n* = 12); the difference between the groups was not statistically significant (*p* = 0.1513). Data are expressed as the mean with SD (one bar per column).

**Figure 3:**
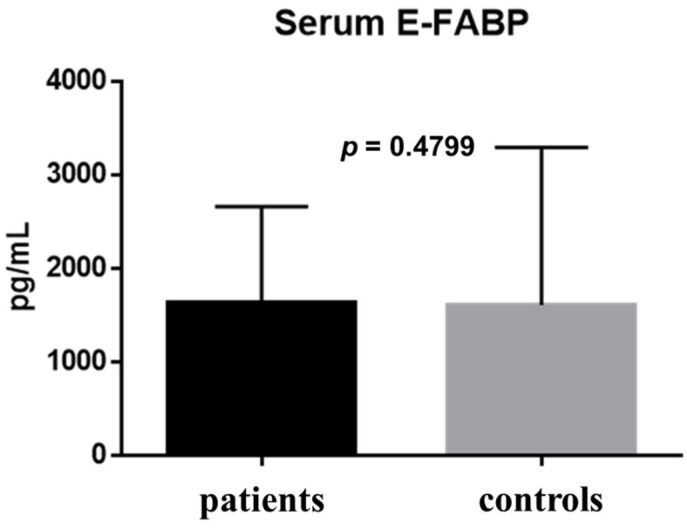
Serum E-FABP concentrations. The E-FABP concentration in the serum was 1630 ± 1030 pg/mL (range: 95.94–3420 pg/mL; median: 1545pg/mL) in the patient group (*n* = 11) and 1604 ± 1686 pg/mL (range: 350.4–6774 pg/mL; median: 1074pg/mL) in the control group (*n* = 12); the difference between the groups was not statistically significant (*p* = 0.4799). Data are expressed as the mean with SD (one bar per column).

The E-FABP concentration in tears was 323.5 ± 325.6 pg/mL in the patient group and 4076 ± 5746 pg/mL in the healthy control group; the difference between groups was statistically significant (*p* = 0.0136) (Figure 1). The E-FABP concentration in saliva was 46.46 ± 38.22 pg/mL in the patient group and 32.08 ± 38.31 pg/mL in the healthy control group (*p* = 0.1513) (Figure 2). The E-FABP concentration in serum was 1630 ± 1030 pg/mL in the patient group and 1604 ± 1686 pg/mL in the healthy control group (*p* = 0.4799) (Figure 3), indicating no significant differences between groups.

The comparison of results between the patients and the healthy control group is shown in Table 2. In the comparison between the patients and healthy control groups, the E-FABP concentration significantly differed in tears only (*p* = 0.0136) (Table 2).

### 2.3. Dry Eye Symptom Questionnaire

The mean dry-eye related quality of life score (DEQS) was significantly higher in the patient group (40.09 pts) than in the healthy control group (7.67 pts) (*p* = 0.001) (Table 3).

### 2.4. Tear Function Examinations

The results of tear break-up time (BUT), ocular surface vital staining, and the Schirmer test-1 are shown in Table 3. The test results were worse in SS patients and significantly differed between the patient and healthy control groups (*p* < 0.0001) (Table 3).

### 2.5. Correlations between Tear Fatty Acid-Binding Protein and DED Parameters

**Figure 4:**
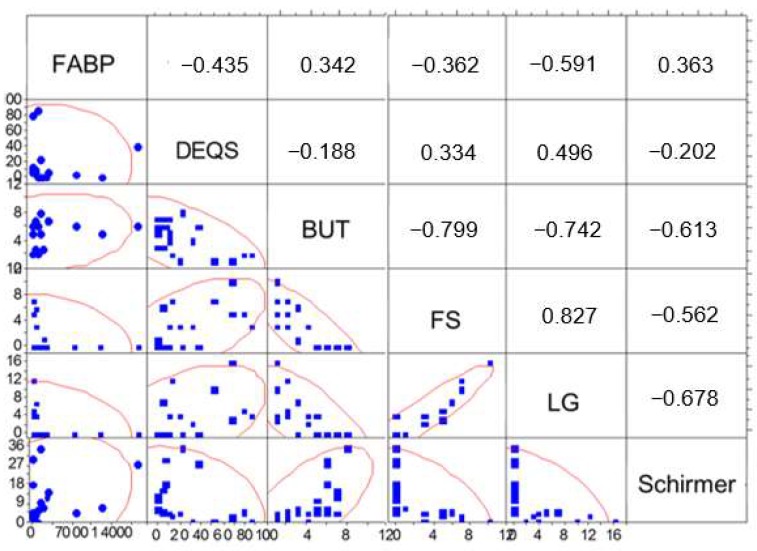
Correlation between tear E-FABP concentrations and DED parameters. *X* and *Y* axis units: FABP: pg/mL; DEQS: points (pts); BUT: seconds (sec); Schirmer: millimeters (mm); FS: points (pts); LG: points (pts).

The results of the Spearman’s rank correlation tests between the E-FABP concentration in tears and the results of the DEQS and tear function examinations are shown in Figure 4. The E-FABP concentration in tears was significantly correlated with the results of BUT (*r* = 0.342, *p* = 0.0179), FS (*r* = −0.362, *p* = 0.016), LG (*r* = −0.591, *p* = 0.001) (Figure 4).

## 3. Discussion

Although the existing diagnostic criteria for SS are continually being revised, improvements can still be made in the areas of early diagnosis and assessment of disease activity. We believe that biomarkers that can be collected using minimally invasive techniques can be used for these purposes. In recent years, many studies have reported the use of the concentrations of proteins in serum, saliva, and tears as a comprehensive screening tool, and these have been predicted to be useful [16,17,18,19,20,21,22]. Five proteins (C-X-C motif chemokine 13 (CXCL13), tumor necrosis factor receptor 2 (TNF-R2), CD48, B-cell activating factor (BAFF), and programmed cell death protein 1 ligand 2 (PD-L2)) related to the disease activity of SS, including the B-cell activating factor (BAFF) [23], which was recently identified by the European League Against Rheumatism (EULAR) as a biomarker that is correlated with the European League Against Rheumatism Sjögren Syndrome Disease Activity Index (ESSDAI) score, have been identified in serum. In addition, previous research assessing the correlation of SS with extraglandular symptoms [24] and research on the S100 protein in tears (which reported oxidative stress damage in cases with SS [25]), have highlighted the possibility that these factors are involved in the mechanism of onset of SS.

E-FABP, which was the focus of our study, has been found not only in epithelial cells, where they are mainly present, but also in a variety of biological tissues other than epithelial cells, such as the brain, liver, and kidneys as well as in adipocytes and in the central nervous system. E-FABP has also been reported to be related to the immune system, oxidative stress, the inflammatory response of tissues, cell differentiation and apoptosis, and malignant diseases [26,27,28,29,30,31,32,33]. E-FABP was originally identified in the skin epithelial cells of rats [26], and therefore, research on this substance in the field of dermatology is advanced. In a study using FABP5 knockout mice (−/−), Owada et al. reported that FABP5 serves an important function in the moisture barrier of skin [33,34]. Possible sources of E-FABP in tears include ocular surface epithelium, meibomian, sebaceous and/or lachrymal glands. E-FABP on the ocular surface might have a similar role as the skin, which is the maintenance of ocular surface epithelial barrier and trans-epithelial water transport. A decrease in E-FABP might lead to disturbances in this barrier, causing increased tear evaporation and dry eyes. In particular, this new marker is known to be related to immunity, and Barbi et al. reported that E-FABP plays an important role in the balance between T-helper 17 (Th17) and T-regulatory (Treg) cell populations [35]. Specifically, A- and E-FABP ligands in the metabolic–inflammatory pathway act as peroxisome proliferator-activating receptor γ (PPARγ) ligands [36], and PPARγ is known to induce the expression of the gene associated with cholesterol transportation [37] and regulate transcription activation of pro-inflammatory genes in macrophages [38,39]. In addition, peroxisome proliferator-activated receptors (PPARs) are adipogenic regulators that have been reported to influence Fabp4 and Fabp5 gene expression [40]. Moreover, in their study of knockout mice, Pan et al. suggested that resident memory T (T_RM_) cells utilize exogenous free fatty acids in the beta-oxidation that occurs in mitochondria, which in turn suggests that FABP4 and FABP5 are involved in increased uptake of free fatty acids [41]. In addition to their role in preventing the infiltration of foreign bodies in barrier tissues, T_RM_ cells have been reported in non-barrier tissues, such as the cranial nervous system, glandular system, lymph tissue, liver, kidneys, pancreas, and the joints, and there is a strong suspicion that they are involved in the pathological expression of chronic inflammatory diseases and autoimmune diseases [42,43,44,45]. T_RM_ cells have also been reported to exist in cases of human dry eye [46]. 

However, in the pathology of SS, the expression of multiple genes that regulate lipid deposition in the lacrimal and salivary glands as well as lipid metabolism has been observed. This is thought to be one major potential area [47,48,49,50,51] in addition to the cross-regulation of the metabolic–inflammatory pathway by FABPs that has the potential to assist in our understanding of the pathophysiology of SS. 

Research on the relationship between SS and FABP includes a study by Baldini et al. which found, through the use of proteome analysis of human saliva, that there was a highly significant difference in FABP5 expression in the SS patient group compared with the healthy volunteer group, and they mentioned the possibility that FABP5 can be used as a specific marker that reflects the disease activity of SS and its severity [52]. It should be noted that the FABP5 concentration in saliva did not differ between patients and controls in our study, differing from the observation in Baldini et al. This discrepancy can be explained by the differences in the systemic disease severity, stage of SS, and the differences in methodology of testing.

Our previous work showed an elevation of lipid peroxidative stress markers in the tears and conjunctiva of patients with SS, concluding that oxidative stress has a role in the pathogenesis of dry eyes associated with SS [8].

In the present study, we compared the E-FABP concentrations in tears, saliva, and serum specimens. To the best of our knowledge, our study is the first to report E-FABP levels in tears. Although we did not find significant differences between the E-FABP concentrations in saliva and serum, we did find that the E-FABP concentration in the tears of patients with dry eye was lower, and in particular, that there was a significant difference between the SS and the healthy control groups. However, it should be remembered that our preliminary results represent 11 untreated patients and further clinical trials on a larger number of subjects will be needed.

The possible advantages of this new biomarker include its detectability in tears by enzyme-linked immunosorbent assay (ELISA), which is a non-invasive method compared to lachrymal gland biopsies. Another advantage is the possibility of the biomarker being an early diagnostic marker, although this needs to be detected in further trials on a large numbers of subjects. The patients in this study underwent initial examination for untreated disease, where our findings may reflect the initial stages of untreated SS in contrast to the patient population in the study by Baldini et al. The current observation, revealing a significant difference of FABP in tears only, suggests that FABP and lipid metabolism in the lacrimal glands and appendages of the eye may be specialized, and also that, r because all the patients in this study were undergoing initial examination for untreated disease, the findings may reflect the initial stages of SS. The finding of correlations between the E-FABP concentration in tears and dry eye parameters suggests that the E-FABP concentration in tears can be used to assess the epithelial damage in the ocular surface, the decline in tear stability and its quality. 

However, there remains a need to investigate the physiological functions of FABP, and in particular, the mechanism of action of FABP on lipid metabolism and the immune system using histological findings obtained from the non-obese diabetic (NOD) and the FABP-KO mice and from further human studies. Our initial work in the NOD SS model mice shows decreased E-FABP tear levels which appear to be correlated with the level of inflammation in tears and the lacrimal gland, and the extent of lipid oxidative stress (data not shown). E-FABP is known to bind to free fatty proteins, downregulating Nos2, Cxcl10 and IL-6 genes causing an activation of NF-κB signaling pathways [53]. Therefore, we expect E-FABP levels to be low when inflammatory pathways are activated. We believe that future investigations to clarify the role of FABP in the pathogenesis of dry eye disease will also lead to the development of novel diagnostic and treatment assessment procedures. The main shortcoming of the current preliminary study is the number of patients which need to be increased in future trials. There is also a need to investigate the E-FABP differences between SS and non-SS or between primary and secondary SS when further research is conducted in the future.

## 4. Methods

### 4.1. Subjects and Examinations

Saliva, serum, and tear specimens were collected from all patients and control subjects, and the E-FABP concentrations in the specimens were measured. Ophthalmological testing consisted of the Dry Eye-related Quality of life Score (DEQS) obtained by a dry eye questionnaire sheet, tear break-up time (BUT) and the Schirmer test-1 for tear function testing, and vital staining with fluorescein (FS) and lissamine green (LG) for ocular surface testing. The study was conducted in accordance with the Declaration of Helsinki and was approved by the Institutional Review Board of Tokyo Dental College (10 February 2016, I 15–89).

### 4.2. Collection of Tears, Saliva, and Serum

Tears, saliva, and serum were collected from the patients in conjunction with the normal tests performed during an initial examination. Twenty microliters of tears (per subject) were collected from the lateral canthus using a glass capillary tube. Saliva was collected when the amount of saliva secretion was measured. Serum was collected using the standard blood collection procedure. The collected specimens were immediately separated by centrifugation at 3000 rpm and 4 °C for 5 min, and the E-FABP concentration in the resulting supernatant was measured by enzyme-linked immunosorbent assay (ELISA). The specimens were stored at −80 °C until analysis.

### 4.3. Enzyme-Linked Immunosorbent Assay

All specimens were thawed, subjected to vortex, and centrifugally separated, and finally, all precipitates were removed prior to assaying. The assays were performed using the FABP5 ELISA Kit (Human) (Aviva Systems Biology, San Diego, CA, USA) based on the standard sandwich ELISA technique and following the manufacturer’s instructions. 

### 4.4. Dry Eye Symptom Questionnaire

The DEQS, (dry eye questionnaire sheet developed in Japan) was carried out in all subjects. The mean DEQS for patients with dry eye and control subjects has been reported to be 33.7 and 6, respectively [54].

### 4.5. Tear Function Examinations

#### 4.5.1. Tear Break-Up Time

Standard BUT measurement was performed. First, 2 µL of a solution with 1% FS (without preservatives) were introduced into the eye with a micropipette. The subjects were asked to blink a few times. Three measurements were taken during the time at which the first corneal black spot appeared after the last complete blink. The mean of these three measurements was used as the tear film break up time.

#### 4.5.2. Ocular Surface Vital Staining

The ocular surface staining scores were assessed using FS and LG dyes. In each case, 2 µL of a solution of 1% FS and 1% LG (without preservatives) was introduced into the eye with a micropipette to stain the subject’s ocular surface. The staining scores were converted to numerals for the purpose of assessment using a 0- to 9-point scale (3 or more points indicates abnormality). 

#### 4.5.3. Schirmer Test-1

The Schirmer test-1, which does not involve the use of a local anesthetic, was utilized. Standardized strips of filter paper (Alcon Inc., Fort Worth, TX, USA) were placed on the lateral canthus of the eye, not touching the cornea, and the subjects were instructed to keep their eyelids closed for 5 min. After 5 min, the length (mm) of the filter paper that was wetted by tears was measured. 

### 4.6. Statistical Analyses

Fisher’s exact test was used to compare the male-to-female ratios in the subject groups (patients and healthy controls), and the Mann–Whitney U test was used to compare age distributions and test results. Spearman’s rank correlation coefficient was used to calculate the correlations between test pairs. A *p* value of less than 5% was considered as statistically significant.

## Figures and Tables

**Table 1:** Revised Japanese criteria for Sjögren syndrome (SS) (1999).

Revised Japanese Criteria for Sjögren Syndrome (SS) (1999)
1. Histopathology Definition: Positive for at least one of (A) or (B): (A) Focus score ≧ 1 (periductal lymphoid cell infiltration ≧ 50) in a 4-mm^2^ minor salivary gland biopsy (B) Focus score ≧ 1 (periductal lymphoid cell infiltration ≧ 50) in a 4-mm^2^ lacrimal gland biopsy
2. Oral Examination Definition: Positive for at least one of (A) or (B): (A) Abnormal findings in sialography ≧ Stage I (diffuse punctate shadows of less than 1 mm) (B) Decreased salivary secretion (flow rate ≦ 10 mL/10 min according to the chewing gum test or ≦ 2 g/2 min according to the Saxon test) and decreased salivary function according to salivary gland scintigraphy
3. Ocular Examination Definition: Positive for at least one of (A) or (B): (A) Schirmer’s test ≦ 5 mm/5 min and rose bengal test ≧ 3 according to the van Bijsterveld score (B) Schirmer’s test ≦ 5 mm/5 min and positive fluorescein staining test
4. Serological Examination Definition: Positive for at least one of (A) or (B): (A) Anti-Ro/SS-A antibody (B) Anti-La/SS-B antibody
Diagnostic criteria: Diagnosis of SS can be made when the patient meets at least two of the above four criteria

**Table 2:** Comparison of tear, saliva, and serum E-FABP concentrations between the patients with SS, non-SS, and the controls. There was a significant difference in E-FABP concentrations only between the SS and the control groups (*p* = 0.0088). * Statistically significant difference by the Mann–Whitney U test.

Subject Subgroups	Tear E-FABP mean ± SD (μg/mL)	Saliva E-FABP mean ± SD (μg/mL)	Serum E-FABP mean ± SD (μg/mL)
SS	89.41 ± 72.30	35.30 ± 24.84	1828 ± 1064
non-SS	674.7 ± 50.73	85.51 ± 63.75	1102 ± 873.3
*p* value vs. SS	*p* = 0.2000	*p* = 0.3333	*p* = 0.4242
*p* value vs. controls	*p* = 0.2857	*p* = 0.1648	*p* = 0.9999
Normal controls	4076 ± 5746	32.08 ± 38.31	1604 ± 1686
*p* value vs. SS	*p* = 0.0088 *	*p* = 0.3075	*p* = 0.3813

**Table 3:** Comparison of dry eye disease (DED) parameters between patients and controls. Statistically significant differences were found between the patient and healthy control groups in dry-eye quality of life score (DEQS) and all ophthalmological tests. * Statistically significant difference by the Mann–Whitney U test (*p* < 0.01). Data are expressed as the mean with SD. BUT: tear break-up time, FS: fluorescein staining score, LG: lissamine green staining score.

Scores	Patients (*n* = 11 eyes)	Controls (*n* = 12 eyes)	*p* Value
DEQS (pts)	40.09 ± 30.66	7.67 ± 11.5	0.001 *
BUT (sec)	2.36 ± 1.43	6.0 ± 1.28	<0.0001 *
Schirmer (mm)	2.73 ± 1.90	14.75 ± 10.73	<0.0001 *
FS (pts)	4.73 ± 2.72	0.08 ± 0.29	<0.0001 *
LG (pts)	6.46 ± 4.39	0.0 ± 0.0	<0.0001 *
